# 3 for the Price of 1: Teaching Chest Pain Risk Stratification in a Multidisciplinary, Problem-based Learning Workshop

**DOI:** 10.5811/westjem.2017.12.36444

**Published:** 2018-03-08

**Authors:** William D. Alley, Cynthia Burns, Nicholas D. Hartman, Kim Askew, Simon A. Mahler

**Affiliations:** * Wake Forest School of Medicine, Department of Emergency Medicine, Winston-Salem, North Carolina; † Wake Forest School of Medicine, Department of Internal Medicine, Winston-Salem, North Carolina

## Abstract

**Introduction:**

Chest pain is a common chief complaint among patients presenting to health systems and often leads to complex and intensive evaluations. While these patients are often cared for by a multidisciplinary team (primary care, emergency medicine, and cardiology), medical students usually learn about the care of these patients in a fragmented, single-specialty paradigm. The present and future care of patients with chest pain is multidisciplinary, and the education of medical students on the subject should be as well. Our objective was to evaluate the effectiveness of a multidisciplinary, problem-based learning workshop to teach third-year medical students about risk assessment for patients presenting with chest pain, specifically focusing on acute coronary syndromes.

**Methods:**

To create an educational experience consistent with multidisciplinary team-based care, we designed a multidisciplinary, problem-based learning workshop to provide medical students with an understanding of how patients with chest pain are cared for in a systems-based manner to improve outcomes. Participants included third-year medical students (n=219) at a single, tertiary care, academic medical center. Knowledge acquisition was tested in a pre-/post-retention test study design.

**Results:**

Following the workshop, students achieved a 19.7% (95% confidence interval [CI] [17.3–22.2%]) absolute increase in scores on post-testing as compared to pre-testing. In addition, students maintained an 11.1% (95% CI [7.2–15.0%]) increase on a retention test vs. the pre-test.

**Conclusion:**

A multidisciplinary, problem-based learning workshop is an effective method of producing lasting gains in student knowledge about chest pain risk stratification.

## INTRODUCTION

Chest pain is a common medical complaint, accounting for 8–10 million emergency department (ED) visits annually.^1^ Health systems care for patients with acute chest pain by using multiple medical disciplines (emergency physicians, primary care physicians, and cardiologists) working as a team. Patients with chest pain typically present to the ED or are seen first by their primary care provider and then sent to the ED. After ED evaluation, low-risk patients are often asked to follow up with primary care, while high-risk patients and those having acute coronary syndrome (ACS) events receive cardiology consultations and are hospitalized for further care. Despite its frequency, the evaluation of patients with chest pain remains complex and nuanced. Although most patients do not have a life-threatening illness, inadvertent discharge of a patient with ACS can result in serious morbidity or mortality.^2^

To avoid missing ACS, while also avoiding over-testing of very low-risk patients, many health systems have adopted objective and multidisciplinary, risk-stratification care pathways.^3^

Given the frequency of chest pain as a chief complaint, third-year medical students have ample exposure to patients with acute chest pain while on their emergency medicine (EM) and internal medicine (IM) clerkships. However, the structure of the traditional third-year curriculum, in which a student rotates within a single discipline and sees a patient through one discipline’s lens, may lead to suboptimal understanding of the patient’s multidisciplinary care. To foster greater understanding of a multidisciplinary, team-based approach to the care of patients with acute chest pain, we developed a multidisciplinary, problem-based learning workshop (MD-PBW).

Prior studies on multidisciplinary education have generally been small, with a focus on measuring the satisfaction of learners and educators.^4^,^5^. While these are important metrics to ensure sustainability of an educational tool, the ability of the tool to deliver and encourage retention of knowledge is at the core of most educational endeavors. In this analysis, we tested whether our MD-PBW increased the medical student’s knowledge of chest pain risk-stratification care and whether they retained this knowledge. We hypothesized that students would demonstrate improved knowledge and would retain a significant portion of this knowledge, as evidenced by scores on pre-tests, post-tests, and knowledge-retention tests, as a result of this educational intervention.

## METHODS

### Study Design

This is a pre-/post-retention test study designed to assess the knowledge acquisition and retention of medical students participating in a MD-PBW focused on chest pain risk stratification. Third-year medical students participated in this study as part of their required IM clerkship. This study was reviewed by the institutional review board of the sponsoring organization and met criteria for exemption based on category 1.

### Population

All participants in this study were third-year medical students enrolled in Wake Forest School of Medicine, an allopathic medical school with an annual enrollment of about 120 students, located in Winston-Salem, NC. These students participated in the educational intervention at varying times during the third-year of medical school, during their required 12-week IM clerkship. This clerkship includes nine weeks of inpatient care, of which two are cardiology. Roughly half of the students had experienced the required four-week EM clerkship and four-week family medicine clerkship prior to their IM clerkship, so presumably would have been exposed to the evaluation of patients presenting with chest pain.

**Population Health Research Capsule**

What do we already know about this issue?

Student and teacher satisfaction for multidisciplinary, problem-based learning workshops (MD-PBW) has been established, but outcomes data testing their effectiveness are limited.

What was the research question?

Does teaching cardiac risk stratification in a MD-PBW produce demonstrable improvement in student knowledge?

What was the major finding of the study?

Results of this study show that cardiac risk-stratification concepts can be effectively taught in a MD-PBW.

How does this improve population health?

Teaching cardiac risk stratification is critical for patient care. Doing so in a multidisciplinary manner reflects the teamwork needed for efficient care.

### Workshop

During the 10th week of their IM clerkship, students participated in two complementary educational events. First was a video presentation, developed in the style of the whiteboard videos made famous by Kahn Academy, viewed by the students detailing the complexities of evaluating patients with chest pain. The video included details of typical and atypical presentations, risk factors for coronary artery disease (CAD), and the use of the HEART Pathway^6^ to risk stratify patients with chest pain.

Following the pre-learn video, students attended a 1.5-hour cardiac risk assessment workshop. During the workshop, students divided into small groups of 8–10 students, each led by three facilitators, with one facilitator from each discipline: EM, general IM, and cardiology. Each small group worked through three simulated patient cases in a PBL-based format. Cases were developed to represent low, intermediate, and high-risk presentations for ACS. Throughout the session, the students were guided by multidisciplinary faculty to organize, synthesize and prioritize the patient’s medical data into an appropriate differential diagnosis and management plan. Facilitators focused on using a multidisciplinary, team-based approach and incorporating objective tools, such as the HEART Pathway,^6^ to more accurately risk stratify patients with acute chest pain.

### Testing

Prior to viewing the animated whiteboard video on chest pain evaluation, students completed a pre-test. Following the MD-PBW, students completed the post-test within one week. One month after the MD-PBW students were invited to complete a retention test. Each test had 10 questions from a question bank. These questions were developed by CB, KA, and SM, and four of the 15 questions were used and showed evidence of validity in a previous investigation by Hartman et al.^7^ Some of the questions on the post-test or retention test were seen on previous tests given during the intervention. Students had 30 minutes to complete each test. Tests were taken electronically using an online testing platform. Students were given a week to take the pre-test and post-test at their own convenience. Retention tests could be taken for up to five months after the MD-PBW.

### Analysis

We analyzed test scores using descriptive statistics, such as mean and standard deviation (SD). Mean percent correct and differences between mean pre-, post-, and retention tests were calculated along with corresponding 95% confidence intervals (CI). To assess for significant differences in test scores we compared the pre-, post-, and retention-test scores using paired t-testing. Statistical analysis was performed using SAS 9.4 (Cary, North Carolina).

## RESULTS

**Figure 1:**
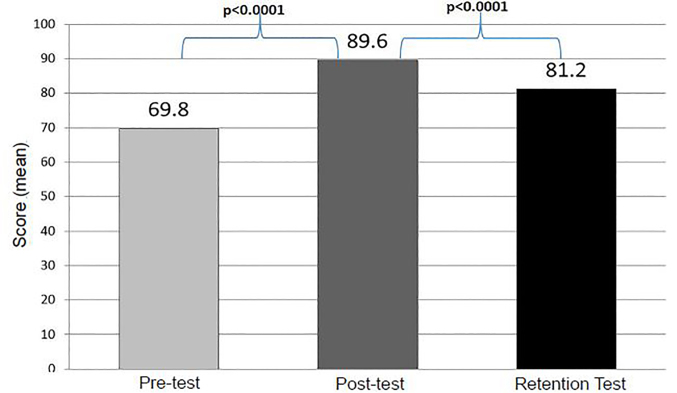
Mean student performance on pre-, post-, and retention test.

From July 2014 to July 2016, 219 medical students participated in the MD-PBW. Among these students, 219 (117 male, 112 female) completed a pre-test, 195 completed a post-test, and 84 completed a retention test. The mean percentage of questions answered correctly on the pre-test was 69.8% (SD 15.7%, 95% CI [67.7–71.2%]), compared to 89.6% (SD 11.4%, 95% CI [88.0–91.2%]) for the post-test, and 81.2% (SD 13%, 95% CI [78.4–84.0%]) for the retention test. Mean test scores are summarized in Table 1 and graphically represented in Figure 1.

**Figure 2:**
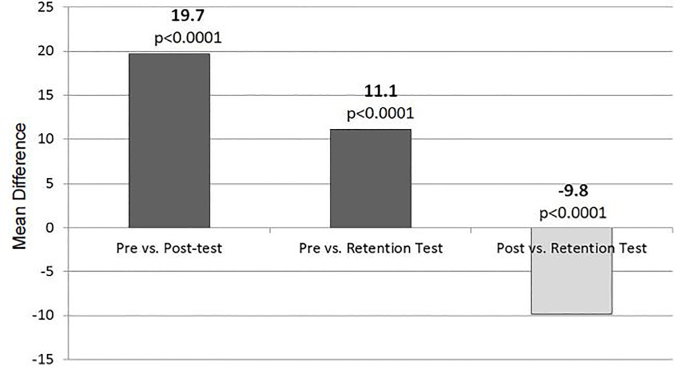
Difference in mean test performance between pre- and post-test, pre- and retention test, and post-test and retention test. Differences in performance all met statistical significance.

Paired comparison of test scores identified 190 students with complete pre-tests and post-tests. Among these students the average increase in score from pre-test to post-test was 19.7 (SD 16.9%, 95% CI [17.3–22.2%]). For the retention portion, 84 students finished both a pre-test and retention test, yielding an average increase in score of 11.1% (SD 17.5%, 95% CI [7.2–15.0%]). Post-tests and retention tests were both completed by 81 students. Among those students, scores decreased by 9.8% (SD 17.6%, 95% CI [5.9–13.6%]). Paired changes in test scores are summarized in Table 2 and graphically represented in Figure 2.

## DISCUSSION

In an ever-changing medical environment, physicians must learn to practice as members of a multidisciplinary team to optimize patient care. Given how commonly patients with chest pain seek care and the relative infrequency with which they are found to have acutely life-threatening disease, it is paramount that budding physicians learn how to make the best use of the available resources within various care settings to optimize outcomes and reduce resource utilization. A focus on service lines and multidisciplinary care to manage these patients has been growing for decades. The number and success of chest pain centers and emergency department chest pain units are prime examples of this trend,^8^,^9^ though education about risk stratification of chest pain is still frequently siloed in a specialty-by-specialty approach. This study aimed to institute and evaluate a multidisciplinary educational intervention to teach students about current practice in risk stratification of patients who present with chest pain.

In our study, medical students demonstrated improved knowledge, both immediately following the intervention as well as up to five months afterward. Variation in the pre-workshop knowledge base of the students is likely related to pre-intervention experience, regarding time in third year as a whole, as well as other clerkships completed. At our institution, third-year medical students have eight required, third-year clinical clerkships, with IM and EM occurring in opposite semesters. This workshop was housed within the IM clerkship, so during the early part of the year, students had fairly limited exposure to the evaluation of patients with chest pain. On the other hand, during the latter half of the year, the majority of students involved in the workshop had completed the EM clerkship, so they were already exposed to the early evaluation of this patient subset. As expected, some of the improvements in knowledge demonstrated by students on the post-test immediately following the workshop diminished over time, as evidenced by performance on the retention test. However, students still had significantly better scores on the retention compared to the pre-test, demonstrating retention of much of the knowledge gained.

These results are promising for a number of reasons. In the changing medical landscape, team-based, coordinated patient care is more and more important. Our results demonstrate that our multidisciplinary, team-based approach to teaching risk stratification of patients with acute chest pain to medical students can produce lasting improvements in knowledge that ideally will translate into better patient care and improved patient outcomes. We believe that a multidisciplinary approach more closely mimics real-world practice, recognizing that patients with chest pain may seek care in a variety of settings, and once they have been initially evaluated and treated, they continue to need evaluation and treatment to ensure that those at high risk are receiving appropriately aggressive care, while lower-risk patients have further evaluation to determine the likely non-cardiac cause of their pain, frequently in the outpatient setting.

We also introduced the students to the HEART Pathway,^6^ a risk-stratification tool used in patients with chest pain concerning for ACS. By including in our discussion a risk-stratification tool designed to focus more resource-intensive cardiac testing and therapies on the patients who are most likely to benefit, students can begin to understand the benefits of more efficient, value-based care. In addition, early education on this topic helps to disseminate information across the potential specialties that the students ultimately decide to pursue, helping to implement multidisciplinary care in a continuous fashion as patients move through initial evaluation and follow-up care.

## LIMITATIONS

This study does have several limitations. First, our study included only a single institution where widespread training for clinical staff on the use of the HEART Pathway as a chest pain risk-stratification tool had already taken place. The implementation and success of a similar educational workshop to the one described here may not be as readily achieved in an institution without a similarly agreed-upon local standard of care. Second, many students did not complete all three tests. This is especially evident in the low completion rate of the retention test. While we believe that the improvements in knowledge are likely representative of the entire group, the possibility of students self-selecting based on how much they remembered of the information taught must be considered. Third, the data do not include a control group, so no comment can be made about whether this MD-PBW is more effective than the traditional teaching model. An argument could be made that a teaching model that more closely mimics the team-based care paradigm being adopted by many health systems to minimize fragmented care is likely still valuable, though further study would be needed to prove this point.

Other studies suggest that similar educational interventions are viewed positively by students.^4^,^5^ No satisfaction data were collected for this MD-PBW. One consideration for future research would be a study that combines satisfaction and effectiveness of a similar intervention in order to better justify the resources required for such an undertaking. It would then be up to a particular institution to decide what amount of educational value would be required to take on an educational offering if it was not viewed positively by students and educators.

Finally, further study would be necessary to determine whether this information was retained long-term. Even more important would be investigation on whether this workshop changed real-time clinical practice and patient outcomes.

## CONCLUSION

A multidisciplinary, problem-based learning workshop increased the knowledge of cardiac risk stratification in patients who present with chest pain among third-year medical students. This builds on previous literature showing increased learner and educator satisfaction with similar educational interventions. Similar MD-PBWs on myriad conditions where a multidisciplinary team approach is beneficial could be used to prepare medical students to provide optimal patient care to improve patient outcomes.

## Figures and Tables

**Table 1:** Mean performance on pre/post/retention tests.

Test phase	Mean	SD	95% CI lower limit	95% CI upper limit
Pre-test	69.8	15.7	67.7	71.9
Post-test	89.6	11.4	88	91.2
Retention test	81.2	13	78.4	84

*SD*, standard deviation; *CI*, confidence interval.

**Table 2:** Difference in performance on testing before and after intervention.

Paired T-test	Mean	SD	95% CI lower limit	95% CI upper limit	P value
Pre vs. Post-test	19.7	16.9	17.3	22.2	<0.0001
Pre vs. retention test	11.1	17.5	7.2	15	<0.0001
Post vs. retention test	−9.8	17.6	−13.6	−5.9	<0.0001

*SD*, standard deviation; *CI*, confidence interval.
